# *Yarsagumba* is a Promising Therapeutic Option for Treatment of Pulmonary Hypertension due to the Potent Anti-Proliferative and Vasorelaxant Properties

**DOI:** 10.3390/medicina56030131

**Published:** 2020-03-16

**Authors:** Himal Luitel, Tatyana Novoyatleva, Akylbek Sydykov, Aleksandar Petrovic, Argen Mamazhakypov, Bhuminand Devkota, Malgorzata Wygrecka, Hossein Ardeschir Ghofrani, Sergey Avdeev, Ralph Theo Schermuly, Djuro Kosanovic

**Affiliations:** 1 Universities of Giessen and Marburg Lung Center (UGMLC), Member of the German Center for Lung Research (DZL), 35392 Giessen, Germany; drhimal@gmail.com (H.L.); Tatyana.Novoyatleva@innere.med.uni-giessen.de (T.N.); Akylbek.Sydykov@innere.med.uni-giessen.de (A.S.); Aleksandar.Petrovic@innere.med.uni-giessen.de (A.P.); Argen.Mamazhakypov@innere.med.uni-giessen.de (A.M.); Malgorzata.Wygrecka@innere.med.uni-giessen.de (M.W.); Ardeschir.Ghofrani@innere.med.uni-giessen.de (H.A.G.); 2 Veterinary Science (Theriogenology), Agriculture and Forestry University (AFU), Center for Biotechnology, 44209 Rampur, Chitwan, Nepal; bhuminand@gmail.com; 3 Department of Pulmonology, Sechenov First Moscow State Medical University (Sechenov University), 119992 Moscow, Russia; serg_avdeev@list.ru

**Keywords:** *Yarsagumba*, cordycepin, pulmonary hypertension, pulmonary vascular smooth muscle cells, pulmonary vasodilatation, biomaterial

## Abstract

*Background and objectives:* Pulmonary hypertension (PH) is characterized by the vasoconstriction and abnormally proliferative vascular cells. The available allopathic treatment options for PH are still not able to cure the disease. Alternative medicine is becoming popular and drawing the attention of the general public and scientific communities. The entomogenous fungus *Yarsagumba* (*Cordyceps sinensis*) and its biologically active ingredient cordycepin may represent the therapeutic option for this incurable disease, owing to their anti-inflammatory, vasodilatory and anti-oxidative effects. *Methods:* In this study, we investigated whether *Yarsagumba* extract and cordycepin possess anti-proliferative and vasorelaxant properties in the context of PH, using 5-bromo-2’-deoxyuridine assay and isolated mice lungs, respectively. *Results:* Our results revealed that *Yarsagumba* extract and its bioactive compound cordycepin significantly attenuated the proliferation of human pulmonary artery smooth muscle cells derived from donor and PH subjects. In isolated murine lungs, only *Yarsagumba* extract, but not cordycepin, resulted in vasodilatation, indicating the probable existence of other bioactive metabolites present in *Yarsagumba* that may be responsible for this outcome. *Conclusion:* Future comprehensive in vivo and in vitro research is crucially needed to discover the profound mechanistic insights with regard to this promising therapeutic potency of *Yarsagumba* extract and to provide further evidence as to whether it can be used as a strategy for the treatment of PH.

## 1. Introduction

Despite the clearly visible progress in the past, pulmonary hypertension (PH) is still an incurable, life-threatening and not completely understood pulmonary vascular disorder, characterized by dysregulated proliferation of vascular cells in the lungs, vasoconstriction, augmented inflammation and severe oxidative stress [1,2,3,4,5,6,7,8]. In recent years, we have tried to change the paradigm in understanding of the pathology of this disease in the context of the Western world, and to suggest new treatment options/possibilities that are derived from traditional medicine of the Eastern civilization [9]. Following this line of reasoning, an interesting entomopathogenic fungus known as *Yarsagumba* (*Cordyceps sinensis* or *Ophiocordyceps sinensis*) represents a promising biomaterial with described beneficial properties for several diseases [10,11,12,13,14]. 

*Yarsagumba* is widely used as a part of traditional medicine in China and it is very popular in several Southeast Asian countries [10]. In nature, this parasitic fungus of the special caterpillar can be found usually in the high altitude mountain regions of Nepal, China/Tibet, Bhutan and India [10,11]. The previous evidence in the literature suggested a plethora of beneficial therapeutic attributes of *Yarsagumba*, including anti-tumor, anti-inflammatory, anti-oxidant, anti-bacterial and vasorelaxant activities or heart protective roles [10,11,15,16].

In recent years, research activities on *Yarsagumba* have attracted great interest in medical science and are gradually increasing. Importantly, the *Cordyceps* has been shown to play a protective role and has provided a promising therapeutic potential in the context of severe lung diseases such as asthma and lung fibrosis [12,17,18,19,20]. It is important to mention that the genus *Cordyceps* contains many bioactive compounds with different biological activities, and one of the best described is cordycepin [21]. Following the previous line, this nucleoside derivative has been also indicated as a potentially anti-asthmatic compound, which reduced experimental asthma by interfering with altered inflammation [22]. Strong anti-inflammatory properties of *Yarsagumba* or cordycepin have been demonstrated in various scientific publications in recent years [23,24]. Furthermore, this natural medicinal entomogenous fungus and its components, such as already mentioned cordycepin, have been investigated for their potent activities against oxidative stress [24,25,26]. Knowing that PH is a disease characterized by dysregulated inflammation, vasoconstriction and oxidative stress, *Yarsagumba* may represent a good candidate for the treatment of this complex pulmonary vascular disease, owing to its above described anti-oxidative, anti-inflammatory and vasorelaxant properties.

The main histopathological characteristic of PH is pulmonary vascular remodeling, which in part is the consequence of abnormally regulated proliferation of the vascular cells. This PH feature is another axis where the probable anti-proliferative effects of *Yarsagumba* and its bioactive components may find a link to this pulmonary vascular disease [27,28,29]. Cordycepin reduced the proliferation of aortic smooth muscle cells, and mechanistically this effect has been explained via decreased expression of cyclins and cyclin-dependent kinase (CDK), induction of G1-phase cell-cycle arrest and upregulation of CDK inhibitor p27^kip1^ [27,28]. In addition to aortic smooth muscle cells, one publication indicated anti-proliferative effects of the *Cordyceps* extract in the context of hypoxia-induced proliferation of pulmonary artery smooth muscle cells [29].

Taken together, the existing evidence indicates that the exact therapeutic potency of *Yarsagumba* and its chemical constituents remain largely unknown in the field of PH. Therefore, the aim of our study is to investigate the potential anti-proliferative and vasorelaxant properties of whole *Yarsagumba* extract and cordycepin.

## 2. Materials and Methods

### 2.1. Preparation of Yarsagumba (Yarsa) Biomaterial

**Figure 1:**
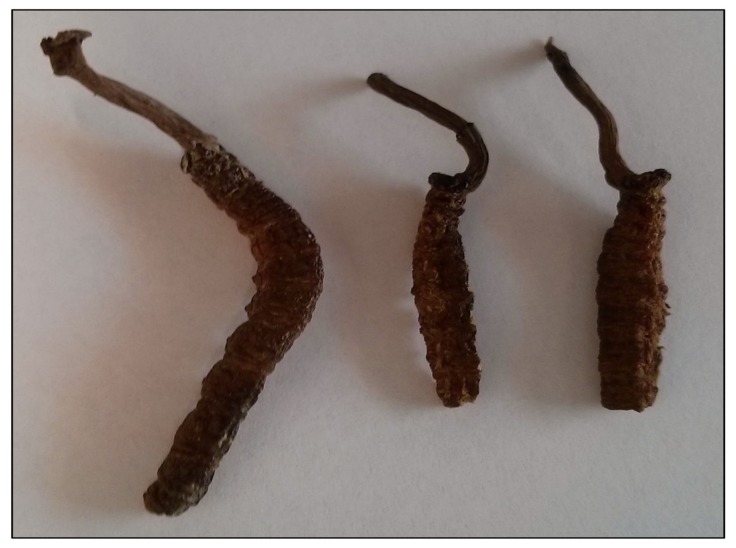
*Yarsagumba*. Intact dried pieces of this entomogenous fungus are presented.

*Yarsagumba* biomaterial is commercially available in Nepal and the formal identification for our study was kindly performed by Dol Raj Luitel from the Department of Plant Resources Office, Ministry of Forest and Soil Conservation, Thapathali, Nepal. First, 50 g of *Yarsa* (Figure 1) was cut into smaller pieces and further crushed by mortar and pestle. The crushed pieces were placed into 2 mL homogenization tubes together with ceramic beads, and filled up with distilled water. Lysis and homogenization were performed repeatedly, by using a Precellys 24 homogenizer (6100, 2 × 45 s, 20 s pause) for 8–10 min in total. After completion of the lysis procedure, centrifugation was performed at 13,000× *g* for 40 min at 20 °C. The supernatant was taken from each homogenization tube and mixed together in one big tube in order to make an equal concentration. The prepared biomaterial (extract) was stored at −20 ˚C until subsequent use.

### 2.2. Ethics Approval and Consent to Participate

Animal experiments were approved by the local authorities (Regierungspräsidium Giessen) and were executed in agreement with the guidelines of the University of Giessen. Human tissue donation was approved by the ethics committee of the Faculty of Medicine at Justus-Liebig University of Giessen in agreement with the principles stated in the Declaration of Helsinki. Written informed consents to participate were obtained from each patient or the patient’s next kin.

### 2.3. Isolated Murine Lungs

In order to investigate the potential effects of *Yarsa* extract and cordycepin (Sigma, C3394, Saint Louis, MO, USA) on hypoxic pulmonary vasoconstriction (HPV), we performed the ex vivo technique of successive hypoxic maneuvers in isolated, ventilated and buffer-perfused mice lungs, similarly to that previously described by our group [3,30]. Initially, C57BL/6 mice (Charles River Laboratories) were anesthetized intraperitoneally with a mixture of ketamine (Ketavet^R^ 10%) and xylazine (Rompun^R^ 2%), followed by removal of the heart and lung for the ex vivo measurements. Mice were kept under standard housing conditions (14/10 h day and night cycle, 55 ± 10% relative humidity, 22 ± 2 °C) in IVC cages in groups of up to five mice per type II long cage. Health monitoring was performed on a regular basis in agreement with the FELASA recommendations. Briefly, cumulative dose–effect curves were created by application of the *Yarsa* extract (volume range: 100–1200 µL) or cordycepin (dose range: 0.08–0.50 mM). Effects of these biomaterials on HPV were compared to the placebo group (vehicle only).

### 2.4. Cell isolation, Culture and Proliferation Assay

Human primary pulmonary artery smooth muscle cells (hPASMCs) of donors were obtained from Lonza (CC-2581, Basel, Switzerland). hPASMCs isolated from patients with idiopathic pulmonary arterial hypertension (IPAH) were derived from our tissue biobank. Both hPASMCs from donor and IPAH were grown in SmGM-2 Bulletkit medium (Lonza). Cells were seeded and grown to 80% confluence prior to treatment. After 24 h of serum starvation, cells were stimulated with SmGM-2 (5% FCS, GM (growth medium)) in the absence or presence of *Yarsa* (volume range: 50–200 µL) and cordycepin (dose range: 0.05–2 mM). Serum-free medium (SFM) was used as a negative control. Then a 5-bromo-2′-deoxyuridine (BrdU) assay was performed after 16 h of stimulation to assess proliferation of hPASMCs.

### 2.5. Data Analysis

All results are expressed as mean ± SEM. Differences between two groups were assessed using Student’s *t*-test. When three or more groups were compared, we used one-way ANOVA followed by Tukey’s post-hoc-test for multiple comparisons. Values of *p* < 0.05 were considered as statistically significant. 

## 3. Results

### 3.1. Yarsa Extract Reduced the Proliferation of hPASMCs

**Figure 2:**
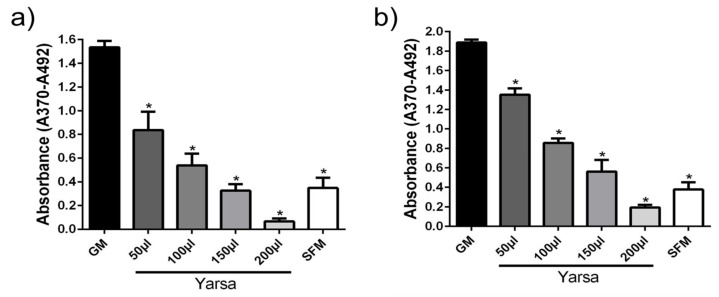
Anti-proliferative effects of *Yarsagumba* extract on human pulmonary artery smooth muscle cells (hPASMCs). hPASMCs were isolated from donors (**a**) and patients with idiopathic pulmonary arterial hypertension (IPAH) (**b**), and cultured. The effect of *Yarsagumba* (Yarsa) extract on cellular proliferation is shown. Results are presented as mean ± SEM (*n* = 3). * *p* < 0.05. GM, growth medium; SFM, serum free medium.

*Yarsa* extract exerted a significant and dose/volume-dependent reduction in proliferation of hPASMCs, as compared to the control (GM; Figure 2). The anti-proliferative effect of *Yarsa* was similar for hPASMCs isolated from donors (Figure 2a) and hPASMCs isolated from IPAH patients (Figure 2b).

### 3.2. Cordycepin Reduced the Proliferation of hPASMCs

**Figure 3:**
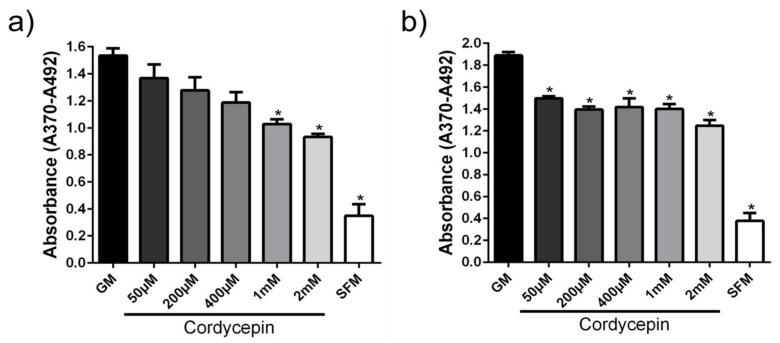
Anti-proliferative effects of cordycepin on human pulmonary artery smooth muscle cells (hPASMCs). hPASMCs were isolated from donors (**a**) and patients with IPAH (**b**), and cultured. The effect of cordycepin on cellular proliferation is shown. Results are presented as mean ± SEM (*n* = 3). * *p* < 0.05. GM, growth medium; SFM, serum free medium.

Similarly, the effect of cordycepin, which is one of the bioactive compounds extracted from the genus *Cordyceps*, on proliferation of hPASMCs isolated from donors and IPAH patients was analyzed (Figure 3). On the one hand, there was a significant and dose-dependent reduction in proliferation of hPASMCs isolated from donors, as compared to the control (GM; Figure 3a). On the other hand, the anti-proliferative potency of cordycepin on hPASMCs isolated from patients with IPAH (Figure 3b) was evident, yet it did not show the clear dose-dependent manner. In general, it seems that the anti-proliferative effects are relatively more prominent in the case of whole *Yarsa* extract, in contrast to the situation when only one bioactive compound, such as cordycepin, was used (Figure 2 and Figure 3).

### 3.3. Yarsa Extract Showed Potent Vasodilatory Properties in Isolated Mouse Lungs

**Figure 4:**
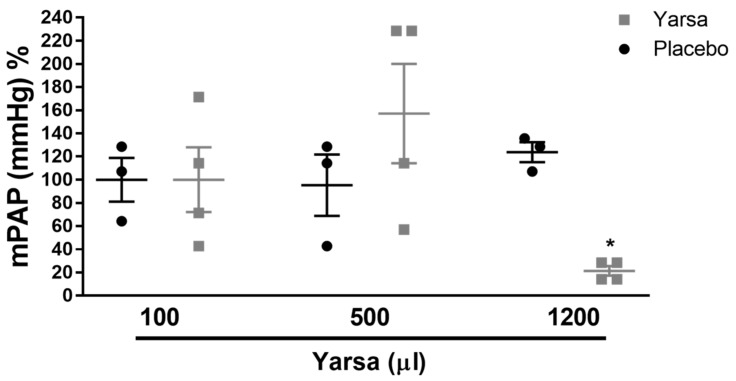
Pulmonary vasodilatory effects of *Yarsagumba* in isolated, ventilated and buffer-perfused mouse lung. Effect of different doses/volumes (volume range: 100–1200 µL) of *Yarsagumba* (Yarsa) extract on hypoxic pulmonary vasoconstriction is shown. Results are presented as mean ± SEM (*n* = 3–4). *p < 0.05. mPAP, mean pulmonary arterial pressure.

The potential vasodilatory effects of *Yarsa* extract were investigated on hypoxic pulmonary vasoconstriction in isolated, buffer-perfused and ventilated lungs of animals (Figure 4). The dose of 1200 µL of *Yarsa* extract exerted a potent reduction in mean pulmonary arterial pressure (mmHg), as compared to the placebo group (vehicle only). 

### 3.4. Cordycepin did not Show any Vasodilatory Properties in Isolated Mouse Lungs

**Figure 5:**
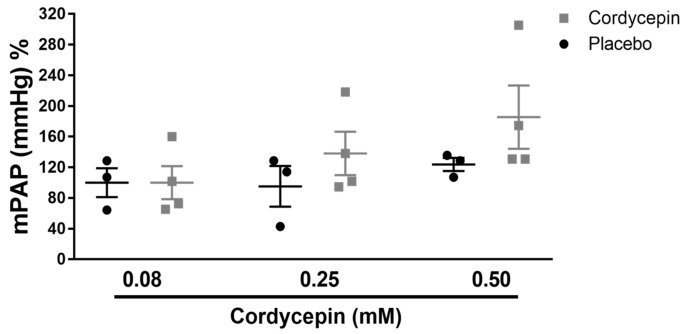
Pulmonary vasodilatory effects of cordycepin in isolated, ventilated and buffer-perfused mouse lung. Effect of different doses (dose range: 0.08–0.50 mM) of cordycepin on hypoxic pulmonary vasoconstriction is shown. Results are presented as mean ± SEM (*n* = 3–4). mPAP, mean pulmonary arterial pressure.

Similarly, the potential vasodilatory effects of cordycepin were investigated on hypoxic pulmonary vasoconstriction in isolated, buffer-perfused and ventilated lungs of animals (Figure 5). Interestingly, this bioactive compound did not reveal any pulmonary vasodilator potency at the tested doses, in comparison to the placebo group (treated only with vehicle).

## 4. Discussion

In general, our findings indicated the following:

(a) The application of the whole *Yarsagumba* extract resulted in significant reduction of hPASMCs proliferation and pulmonary vasodilatation in isolated, buffer-perfused and ventilated mice lungs.
(b) With regard to cordycepin, an active biochemical agent isolated from *Yarsagumba*, there were also significant anti-proliferative effects on hPASMCs; however, the effects were less prominent in comparison to the whole extract of this parasitic fungus. Finally, there was no visible effect of cordycepin on pulmonary vasoconstriction.

Different literature sources indicated that the application of *Yarsagumba* or its bioactive substance, such as cordycepin, exert anti-proliferative effects on aortic smooth muscle cells, as well as on the rat PASMCs [27,28,29]. In agreement with the literature, our findings clearly demonstrated that *Yarsagumba* extract significantly decreased the proliferation of human PASMCs in donors as well as in patients suffering from IPAH. Furthermore, we revealed that such an effect was volume-dependent. Keeping in mind that the altered proliferation of PASMCs is a characteristic of a pulmonary vascular remodeling process, our data clearly suggest a potential future use of this medicinal fungus in the context of PH. Future experiments are needed to investigate the potential effects of *Yarsagumba* and cordycepin on other relevant cell types, such as endothelial cells and fibroblasts.

Imbalance of pulmonary vasoreactivity towards the direction of vasoconstriction has been a well-described pathological event in PH [1]. It has been shown that the extract from the genus *Cordyceps* provided a vasorelaxant response in systemic circulation [10,16]. In the case of pulmonary circulation, our results revealed that *Yarsagumba* extract also demonstrated potent vasodilatory properties. Surprisingly, cordycepin alone did not show any effect on hypoxic pulmonary vasoconstriction. 

Following our observations that the whole extract of this fungus–caterpillar biomaterial exerted more prominent anti-proliferative effects in comparison to its purified bioactive compound cordycepin, and knowing that the whole *Yarsagumba* extract decreased pulmonary vasoconstriction, while cordycepin alone did not show any effect, it is tempting to speculate that the promising therapeutic effects may be expected to result from the combination of various bioactive agents, rather than one single compound alone. However, future research should reveal whether this is the real fact. Many different compounds in this traditional medicine have been already identified; however, their exact biological functions and the completion of the list of such “health beneficial biochemicals” derived from *Yarsagumba* are still not fully established [10,11].

**Figure 6:**
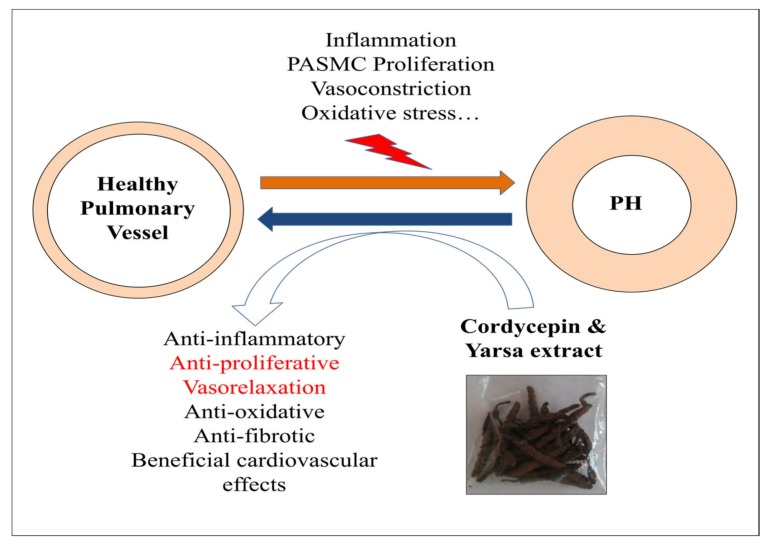
Hypothetical mechanisms and promising future therapeutic properties of *Yarsagumba* and its bioactive compound cordycepin in the pathology of pulmonary hypertension. PH, pulmonary hypertension, PASMCs, pulmonary artery smooth muscle cells, Yarsa, *Yarsagumba*. The black text indicates the findings from the literature and red text indicates the contribution of the current study [9,10,11].

In addition to the reduced PASMCs proliferation and promising vasodilatory potency, and based on several experimental studies, *Yarsagumba* may be expected to provide further anti-PH effects due to its anti-inflammatory, anti-oxidant, cardiovascular beneficial and anti-fibrotic properties (Figure 6) [12,15,17,18,19,22,24,25,26]. There is one important limitation of the present study. The potential therapeutic effects of *Yarsagumba* and cordycepin should be investigated using different in vivo models of the disease in the future, such as chronic hypoxia-induced PH, and monocrotaline and hypoxia + SU-5416 models of PH.

## 5. Conclusions

In conclusion, we have demonstrated for the first time that usage of *Yarsagumba* extract may be a promising therapeutic strategy for the treatment of severe and incurable disease, such as PH, due to its promising anti-proliferative and vasodilatory characteristics. Future profound in vitro studies are needed to exactly identify the underlying molecular mechanisms that may be responsible for reduction of human PASMCs proliferation and pulmonary vasodilatation. Finally, comprehensive in vivo studies on different animal models of PH are crucially required in order to investigate the potential effects of this traditional Eastern medicine in chronic disease models.
